# Fruit flies on the front line: the translational impact of *Drosophila*

**DOI:** 10.1242/dmm.024810

**Published:** 2016-03-01

**Authors:** Norbert Perrimon, Nancy M. Bonini, Paraminder Dhillon

**Affiliations:** 1 Department of Genetics, Harvard Medical School, 200 Longwood Avenue, Boston, MA 02115, USA; 2 Biology Department, University of Pennsylvania, Philadelphia, PA 19104, USA; 3 Disease Models & Mechanisms, The Company of Biologists, Bidder Building, Station Road, Histon, Cambridge, CB24 9LF, UK

**Keywords:** *Drosophila*, Cancer, Genomics, Neurodegeneration

## Abstract

*Drosophila melanogaster* has been adopted as one of the most-used model systems since it was first introduced by Thomas Morgan for the study of heredity in the early 20th century. Its experimental tractability and similarity of its biological pathways to those of humans have placed the model at the forefront of research into human development and disease. With the ongoing accumulation of genetic tools and assays, the fly community has at its fingertips the resources to generate diverse *Drosophila* disease models for the study of genes and pathways involved in a wide range of disorders. In recent years, the fly has also been used successfully for drug screening. In this Editorial, we introduce a Special Collection of reviews, interviews and original research articles that highlight some of the many ways that *Drosophila* has made, and continues to make, an impact on basic biological insights and translational science.

## Abstract (precis)

***Drosophila* Collection:** This Editorial introduces a new Special Collection, ‘Spotlight on *Drosophila*: Translational Impact’, providing a summary of its contents so far and highlighting the impact of *Drosophila* as a model for human diseases.

## Why fly?

Identifying functions for each of the 20,000-25,000 estimated human genes, and determining which of these genes play a role in disease, is one of the greatest challenges in medicine today. Although the advances in genome sequencing over the past decade have meant that DNA variants linked to disease can now be rapidly and accurately identified, understanding whether a particular variant is causal and, if it is, how it perturbs cellular function and overall organismal physiology remains a daunting problem. The major obstacles to determining causality and understanding underlying pathogenic mechanisms are: (1) in the majority of cases the cellular function of a specific gene is unknown, meaning that we lack hypotheses to link the disease to a physiological process; (2) genetic diseases are commonly the products of mutations in multiple genes in combination with lifestyle and environmental factors; and (3) organisms are complex integrated systems within which there is extensive cell- and tissue-level communication; thus, understanding the basis of human syndromes requires a systems biology approach.

Analysis of the full *Drosophila* genome, completed in 2000, and recent further annotation by the modENCODE consortium reveal striking conservation between fly and human genes, underscoring the utility of *Drosophila* for studying fundamental processes relevant to human health. Indeed, *Drosophila* orthologs have been identified for approximately two-thirds of all human disease genes, and all major signal transduction pathways are conserved between flies and humans (Perrimon et al., 2012). Importantly, many of the internal organ systems of *Drosophila* are functionally analogous to those in humans: the fly has a beating heart, sophisticated musculature, a tubular network analogous to lungs, an osmoregulatory/excretion system analogous to kidneys, adipose tissue that functions as the equivalent of the liver, a complex brain and nervous system with glial helper cells and protected by a barrier akin to the blood-brain barrier, and many other parallels. Finally, the extensive and ever-growing genetic toolkit allows precise spatiotemporal control of gene expression and perturbation, and sophisticated genome engineering. The fly is thus poised to be a key player in ongoing efforts to functionally characterize all the disease-associated variants that are being unearthed by genomic analyses.

**Figure:**
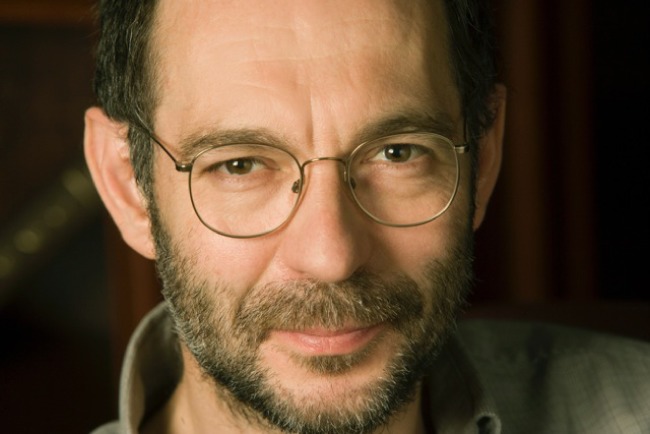
Norbert Perrimon

Our goal in compiling this special Disease Models & Mechanisms (DMM) collection, ‘Spotlight on *Drosophila*: Translational Impact’, is to illustrate the many ways that *Drosophila* can be used to understand and provide the foundation for treatments for human diseases. To launch the collection, we present, in this issue, a number of new reviews and research articles that exemplify some of the insights that have been obtained from modeling human diseases in the fly. We also highlight some of DMM's ‘best’ fly-focused research and review content from recent years. Below, we summarize the collection and provide a flavor of what's to come in upcoming issues of the journal.

## Frontrunners: reviews and more

First, members of Hugo Bellen's lab present a visual overview of the *Drosophila* tools and assays that can be utilized to generate fly models of human diseases and study disease-associated genes. In their ‘At a Glance’ poster article, they highlight how, despite the morphological differences between some fly and human organs, the extensive toolbox available for *Drosophila* research can provide unique insights into mechanisms of diseases affecting the central nervous system, heart, liver and kidney (Ugur et al., 2016). This broad theme, of *Drosophila* studies yielding successes in modeling diseases, is further emphasized by Matthew Moulton and Anthea Letsou, who focus their Review on what has been learned from using the fly to model congenital disorders or inborn errors of human development (Moulton and Letsou, 2016).

Next, Milburn et al. describe a recently added feature of FlyBase, the model organism database for *Drosophila*. They illustrate, with a specific disease example, how the introduction of integrated Human Disease Model Reports to FlyBase will improve the availability and accessibility of fly data relevant to human diseases (Millburn et al., 2016). Their ongoing efforts to curate the data and make the information readily searchable and user-friendly will promote collaboration within the *Drosophila* community and, crucially, also with other communities in which similar questions are being addressed using complementary models and approaches.

**Figure:**
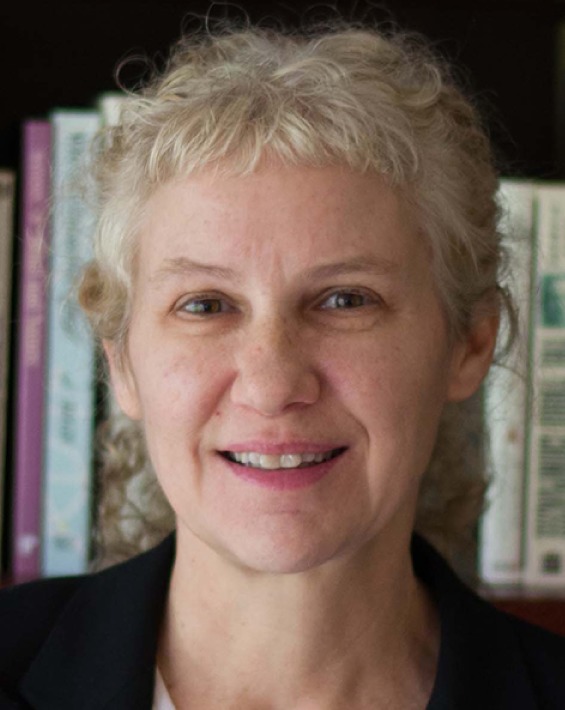
Nancy M. Bonini

It is now well established that the major metabolic, energy-sensing and endocrine signaling networks of *Drosophila* and vertebrates share many similarities. In particular, flies have insulin, glucagon and leptin hormonal systems that regulate metabolic homeostasis and nutrient-sensing. In a previous issue of DMM, Owusu-Ansah and Perrimon discussed the endocrine mechanisms that regulate carbohydrate and lipid metabolism, and the various efforts at modeling the effects of high-fat or -sugar diets in *Drosophila*, as well as the signaling mechanisms involved in integrating organ function (Owusu-Ansah and Perrimon, 2014). In the next issue, Ron Alfa and Seung Kim will review how defects in these pathways lead to phenotypes that are analogous to diabetic states in mammals, and how the fly can be used to characterize the role of potential type-2 diabetes-mellitus-associated genes in insulin-secretion or insulin-resistance pathways (Alfa and Kim, 2016).

Studies of metabolism in the fly must also address the involvement of microbiota, given that flies, just like vertebrates, need to interact productively with commensal microbes while combating pathogenic microbes. Wong et al. review the impact of both commensal and pathogenic intestinal microbes on *Drosophila* carbohydrate and lipid metabolism, and how these studies are contributing to the identification of metabolites produced by intestinal microbes, the intestinal receptors that sense these metabolites, and the host signaling pathways modulated by these metabolites (Wong et al., 2016). Coming up in a future issue of DMM, Hongjie Li and Heinrich Jasper will touch upon aspects of microbe-host interaction in the context of their review focused on the link between intestinal stem cells and human diseases, particularly cancer.

*Drosophila* is also a well-established model for aging, not least because flies have a lifespan of around 3 months. The roles of many genes, pathways and cellular processes (e.g. oxidative stress, hypoxia, mitohormesis, proteostasis, DNA damage) that affect the aging process have been elucidated in the fly. Because aging is an organismal process, studies in *Drosophila* allow dissection of both tissue-intrinsic and -extrinsic factors, as discussed in another earlier review, written by Fabio Demontis and colleagues, on the mechanisms of skeletal muscle aging and the relevance to sarcopenia (Demontis et al., 2013).

## Research in flies: from neurodegeneration to cardiovascular disease

In addition to the diverse review content, a number of original research articles are showcased in our Special Collection. Several articles present findings pertinent to understanding neuronal function in the context of neurological and neurodegenerative diseases, illustrating how the fly is particularly well-suited for the exploration of central nervous system disorders.

In the first of four new research papers published in this issue, a team led by Doris Kretzschmar used a series of elegant assays to demonstrate that glia-specific knockdown of *swiss cheese* (*SWS*), the *Drosophila* ortholog of human neuropathy target esterase (*NTE*), impairs neuronal function (Dutta et al., 2016). Their results shed new light on the pathology of NTE-associated spastic paraplegia, for which the role of glial-specific defects had not yet been explored. The study also contributes to our understanding of neuron-glia interactions in disease – an exciting area of research that has recently burgeoned.

Also in this issue, Kyoung Sang Cho and colleagues provide evidence that upregulation of the *Drosophila sra* gene augments neuropathogenic symptoms in a fly model of Alzheimer's disease (AD). The protein encoded by the human homolog of *sra*, known as Down's syndrome critical region 1 (DSCR1) protein, is a calcineurin inhibitor whose levels are elevated in the brains of individuals with AD (Lee et al., 2016). However, whether the protein conveys a pathogenic or neuroprotective role in the disease had been debated. This new study suggests that upregulation of the protein exacerbates pathogenesis.

In a third newly published study, Anne-Laure Bouge and Marie-Laure Parmentier use the *Drosophila* wing epithelium as a model to examine the effects of excess Tau on mitosis. Tau is a microtubule-binding protein that has been implicated in several neurodegenerative diseases, including AD. A hallmark feature of AD is aneuploidy, and it had been postulated – but not verified – that mitotic defects driven by excess Tau in the brains of affected individuals could be a contributing factor. Now, Bouge and Parmentier (2016) show that an excess of human Tau in dividing cells causes mitotic arrest in flies, correlating with the presence of monopolar spindles, aneuploidy and cell death. They also give insight into the mechanism of action, involving inhibition of kinesin-5. These findings provide evidence of a causal link between aneuploidy and excess Tau that might contribute to AD.

Further illustrating how *Drosophila* research has contributed significantly to studies of AD, many other insights into the mechanisms underlying the complex pathogenesis of this neurodegenerative disorder have been reported recently in DMM. In two separate articles, published in 2014 and 2015, Damian Crowther and colleagues used transgenic flies expressing the amyloid beta peptide (Aβ) to provide insight into circadian behavioral defects and the role of iron in this AD model (Chen et al., 2014; Ott et al., 2015). As another example, the Marenda lab used the *Drosophila* larval neuromuscular junction to examine synaptic dysfunction in their recently generated model of AD based on expression of the human AD-associated proteins APP and BACE (Mhatre et al., 2014).

The fly neuromuscular junction was also used as a model by Thomas Raabe and colleagues, this time to study the X-linked disorder Coffin-Lowry syndrome (CLS). They showed that removal of the fly ortholog of human *RSK2*, a regulator of ERK signaling, leads to defects in synaptic function and anterograde axonal transport in *Drosophila* motoneurons (Beck et al., 2015). Although the postsynaptic function of *RSK2* had been indicated in previous work using animal models of CLS, this study uncovered a novel presynaptic role for the regulator, demonstrating how flies can bring to light unexpected and unsuspected roles for known genes.

Flies are also highly amenable to a range of cardiovascular assays, as illustrated in the collection's poster article and exemplified by a recent DMM Research Article. Ruben Artero applied semi-automatic optical heartbeat analysis (SOHA) to assess heart function in their newly generated fly model of myotonic dystrophy type 1 (DM1) cardiac dysfunction. Mimicking what is observed in humans with DM1, these flies show reduced survival, increased arrhythmia, altered systolic and diastolic function and reduced contractility. Providing proof-of-concept that the model could be used as an *in vivo* platform to evaluate potential therapeutic compounds, the authors also reported the efficacy of a known anti-DM1 drug, pentamidine, to effect partial rescue of the heart phenotypes (Chakraborty et al., 2015).

## Concluding remarks

In recent years, the world of fly models for human disease has exploded. We anticipate continued growth in this area as advances in genomics lead to a greater need to understand the impact of polymorphisms in genes, as well as other influences on disease, including environmental triggers. *Drosophila* models allow elucidation of the biological pathways and environmental factors that influence the clinical presentation of the disease, providing a wealth of information for translational efforts in compound and small-molecule screening.

This Collection also highlights the creativity and innovation of the fly community in generating important and appropriate models for a range of human biological complexities. We also underscore the collaborative and interactive spirit of the fly community as a whole to advance *Drosophila* research, a theme that is discussed by Gerry Rubin in his interview with DMM, coming up soon. The sharing of reagents is a fundamental backbone of these interactions, as are the advances in technological approaches to manipulate gene function in the animal in ways that allow detailed study and exquisite understanding of pathways and the impact of their disruption. These features are among those that have allowed *Drosophila* studies to take a prominent position in providing insight into human biology and disease. We hope you will enjoy the Collection, which will be added to over the coming months, with all articles being collated on this dedicated page: http://dmm.biologists.org/collection/drosophila-disease-model.

We dedicate this Collection to Marcos Vidal, who sadly passed away in January, 2016. An obituary written by Ross Cagan and Eyal Gottlieb is also published in this issue (Cagan and Gottlieb, 2016).
